# Mechanical Dentistry

**Published:** 1877-10

**Authors:** C. B. Rohland

**Affiliations:** Alton, Illinois.


### ARTICLE IV.

### *Mechanical Dentistry.*

BY C. B. ROHLAND, ALTON, ILLINOIS.

Probably no where else do we see the principles of Æsthetics more frequently violated than in Mechanical Dentistry. It is comparatively an easy matter to construct a piece of dental mechanism which will serve the purposes of speech and mastication, but to so construct it as to close the gap which disease or accident may have created, so as to appear as though it really belonged there—from the infrequency with which it is done, the average observer would naturally infer it to be either very difficult, or require qualities of head and hand seldom met with in the profession. The laws of harmony and the paramount necessity of observing them in Dental Prosthesis, have frequently been discussed. Scarcely anyone but knows that the size, shape, color and expression of the teeth should be but complemental to the size, shape, color and expression of the face; that an artificial substitute, must contribute to and maintain the individuality of the patient, and not destroy or even modify it; that to secure the highest expression of the art, all incongruous lines, forms and shades must be avoided and the substitute, not literally but in effect, be part of the patient, bone of his bone and flesh of his flesh. And yet how seldom we see the full realization of these principles. How often the educated eye detects the most inharmonious jumbling together of parts. It has been estimated that not more than one in five hundred artificial dentures made could be safely considered a true expression of high art. A bold estimate certainly, but one that I hardly think we are justified in entirely ignoring. To acknowledge this however, is in no wise a reflection on either the honesty, intelligence or progressive spirit of the profession. It is not my purpose therefore to assume the role of a modern Jeremiah, bewailing and lamenting over Mechanical Dentistry as one of the lost arts, or to utter senseless philippics on the degeneracy of the present. The ghost of the dead past can point to no acquirements that the living present cannot discount.

If we allow ourselves to be influenced by the lamentations of those who have been so liberally berating the profession for its apparent lack of progress in this branch of the art, we will be apt to do ourselves and our calling gross injustice. I use the terms, "apparent lack of progress," advisedly, and I wish to take exception to the assumption that Mechanical Dentistry has not advanced, but on the contrary, retrograded. I wish to add a gentle minor of dissent to the grand chorus of lamentation with which we have been so liberally favored. If we as a profession have so improved our materials and methods of work, that where one formerly was able to avail himself of the benefits of our art we are now able to add to the comfort and happiness of hundreds, why this, I call progress. And this, I assert, we have done. If the knowledge, taste and experience which formerly was confined to very few, is now diffused among the profession generally, and this supplemented by improved materials and appliances, this too is progress. I am now speaking of the profession generally, and not of isolated cases. Then again, if what formerly required exceptional skill to accomplish but indifferently well, has been so simplified that the same object is accomplished in less time, and with less trouble and expense, then this, too is progress. And this has been done. That new methods require less manual skill, is no evidence of retrogression. The labor-saving and time-saving machinery which crowds our mills and factories, have not the less developed our industries, and are not the less emblems of progress that have contributed to the comfort and happiness of the world because forsooth, they superseded manual skill.

Our mission, as Mechanical Dentists, is to restore the lost or impaired functions of speech and mastication, and preserve as near as we can, the features and expression of the human face. And will any one deny that we, as a profession, fulfill our mission better now than formerly? The artificial dentures of to-day serve their purpose better than the artificial dentures of yesterday. To assume the contrary is to assume that the profession of the present is not as honest, intelligent and painstaking as the profession of the past; that trickery, fraud and cupidity are characteristic of the Mechanical Dentist of to-day. That, in other words, he pursues his calling not for the good of his fellow man, but from the most sordid and selfish of motives, and fails to profit from the experience of the past, through wilful ignorance or indolence. What an absurdity! What rank injustice! Our knowledge of and acquaintance with the many true, earnest and intelligent men composing the bulk of our profession, forbids accepting such an assumption. Our calling has its rump adherents it is true, but the profession proper is composed of just as honest, conscientious, intelligent, and earnest men as are to be found anywhere. In these qualities it stands second to no other in the world. Conceding this then, does it not stand to reason, that with the increased knowledge and experience, and the improved methods and materials at our command, we should have advanced, rather than retrograded? Much indeed, is still to be learned, and more perhaps, unlearned. Perfection is still in the dim distance, but we have every reason to believe that we are approximating it. The main obstacles in our path are, not the lowered standard of intelligence and morality, but the imperfection of the materials at hand with which to work.

It has been well said that nothing of human invention is ever perfect, and this is especially applicable to the materials used in the construction of Artificial Dentures. The greater the skill and taste of the dentist, the more he feels himself limited and baffled at almost every step, in giving them true expression. On the very threshold of his attempt to furnish a substitute, fulfilling all the requirements of Æsthetic Dentistry, practical use, and the daily exigencies of an average practice, he finds hisefforts cramped on all sides.

What then is mainly required to construct a perfect set of teeth? Let us examine with the lights we at present have. Assuming the perfect set to be constructed on the same general principles as at present, we would require a base thin and light as paper, flexible enough to adapt itself perfectly to all the variations of the mouth in form and movement, yet firm enough to serve the purposes of mastication; indestructible; perfect conductor of thermal changes; perfectly transparent, or of natural gum color; and last but not least, easily manipulated, and comparatively inexpensive.

Such a case, combined with plain teeth a trifle more durable, with bicuspids and molars a trifle less impossible in shape than most of those at present furnished, would perhaps come as near enabling the skilful artist to put his feelings into shape as anything we can now conceive of within the bounds of possibility. This is what we would like to have and what we have been striving for, and this is what we still fail to grasp. A moment's consideration of the various bases now in use, while demonstrating the onward movement of Mechanical Dentistry, will also show some fatal defect in each. Gold, Continuous Gum, Porcelain, Aluminum, Rubber, Celluloid, or their combinations, each having points of merit, all fail in some important particular. While Continuous Gum and Porcelain Base probably fulfill the requirements of Æsthetic Dentistry better than any of the others, they are, as at present consti tuted, practically out of the question as substitutes for the Perfect Base. First, on account of the difficulty of manipulation, and second, on account of the popular demand. Not that it requires too much taste and skill, because the profession has grit and ability enough to wring success out of anything that gives fair promise of supplying its needs, but because its results are not commensurate with the time, trouble and labor involved, with the demands it makes on the patience of the dentist, and the time of his practice. This is an actual fact in actual practice, and captious criticisms will not make it any the less a fact.

The demands of Operative Dentistry are increasing year by year, and with the increased attention to the preservation of the natural teeth follows a naturally decreased demand for the services of the Mechanical Dentist, so that where formerly three-fourths of an average practice was purely mechanical, the exact reverse of this is true at present. In our estimate then of the availability of a base, this fact must enter largely into our calculations, and will in a measure explain why these bases have not been more generally adopted. Add to this the demands of the patients themselves, and we have a combination of obstacles which practically render useless these undoubtedly meritorious bases. Of course, we should educate the public! Of course! We have been told that so often, that it necessarily must, or at all events should be, just the thing. Well! We see enthusiastic youths rushing forth from our schools and colleges every year, animated with the noble and glorious purpose of educating the world, and generally winding up by—being educated! And this is about the result in practice. Your ordinary public is an obstinate beast to educate, and the task is rendered ten times more difficult if it makes what it conceives to be additional inroads on its pocket. The dentist with the average practice who insists on regulating the demands of his patients and banishing cheap bases entirely, enters upon an unequal contest; for while persistent effort may occasionally bear some fruit with those who demand the cheaper bases from choice, back of them come the hosts of those who ask them from necessity, and to whose comfort and happiness we are just as much bound to contribute. Theoretically, the former ought to know better, and if not they should be taught, but practically they do not know better, and have a beastly way of going somewhere else to get what they want, if one undertakes to educate them too much. They cannot see the use of expending a hundred dollars for an artificial denture when their neighbor eats, talks, and swears with old-time volubility, with a set just out of a steam tooth factory for eight dollars. They have an idea they are doing very well in being willing to pay a regular dentist two or three times as much for a denture that certainly will not, as far as their comprehension goes, accomplish more for them. With the latter, and their name is legion, who demand cheap bases from necessity, of course we cannot quarrel; our plain duty is to do the best we can for them under the circumstances. And here let me remark, *en passant*, in answer to certain strictures frequently made on the profession, that while an honest dentist can never be persuaded by those who put themselves under his care to do anything which he is satisfied is wrong, and will result in their material injury, yet in the matter of artificial dentures, after giving them the benefit of his best judgment and advice, he has no more right to abuse them for making a choice in accordance with their feelings or necessities, than "The Butcher, the Baker, or the Candle Stick Maker" has to quarrel with the choice of his customers.

Here then are some of the conditions under which nine-tenths of the dentist, in this country are practicing, and here is to be found the key to the stronghold that rubber and "its equivalents" have taken upon the profession. When first introduced, it gave promise of supplying a long felt need. Although comparatively ignorant of its properties, the profession took hold of it, and wrought with it, until it compassed its possibilities and made it supply, though indifferently well, the place of a cheap and useful base for artificial dentures. There must have been something good something just needed in it, to withstand the bitter abuse of which it was made the subject. To denounce it was a badge of honor, to countenance it was to be ranked with the rump adherents of the profession. The secret of all this bitter opposition to it, aside from the tax on its use, was not so much its imperfections, but the fact that its easy manipulation opened the doors of Mechanical Dentistry to hundreds who, whatever their pretensions, were unable to do anything else than—spoil rubber. This was an evil of course, but could not be remedied by declining to avail ourselves of its advantages. Because quacks are the better enabled to pursue their calling by the introduction of simplified methods and materials, is no reason why we should refuse to adopt them. By the way, does it not seem a little singular that the profession while vigorously denouncing rubber, as vile trash, a curse, and the invention of the devil generally, should at the same time have just as vigorously fought for its free use! That the same societies and organizations that passed resolutions denouncing its use, and those countenancing it, should afterwards adjourn and form themselves into a Holy Alliance, sworn to fight the tax on its use, until the last dollar they could collect was spent! Was not that just the least bit inconsistent?

The advent of rubber, with its advantages and disadvantages, turned the attention of the profession in a new direction, and various spasmodic attempts at improvement finally blossomed forth into celluloid. This last introduction represents the striving after better things; it is, apparently at least, one step nearer the perfect base; light, indestructible in the fluids of the mouth, harmless, tolerably good color, easily manipulated, and comparatively inexpensive, it only lacks improved durable color, slight additional strength and toughness, so as to permit its being made thin enough to be flexible, and the test of time, to make it take a position far in advance of anything yet offered to the profession. It certainly seems to have the advantage of rubber in every respect—in beauty, cleanliness, healthfulness, color, close adaption to the teeth, and certainty of results in manipulation. Its durability alone, seems still to be in question; but if a denture of celluloid in constant use for three or four years shows no signs of disintegration, one is justified in expecting it to last three or four years longer, and this after all, is just about the average life of the rubber plate. If formerly, under the use of rubber, ten plates came back for repairs to one now under the use of celluloid, is not one almost justified in anticipating the test of time? And this gentlemen, has been my experience. As a substitute for rubber, uniform success, so far, has been the rule in my use of it? Indeed, so certain have the results been, that where failures have occurred they have invariably been due to accident, carelessness, or bad judgment. Whether my standard of excellence is not so high as that of Drs. Chase, Welchens, and a few other careful and intelligent observers who have expressed their disappointment in their use of this base, I do not know. I confess this discrepancy in experience has puzzled me considerably; I cannot account for it; I have no new or unusual methods of working it; I only know that in my practic, celluloid satisfies the requirements of healthfulness, comfort, and æsthetics, in all cases better than rubber, and in a great many cases better than the old style metal plate. One defect however in a majority of cases, is a gradual change of color, varying with the habits of the patient, and the conditions of the mouth. Whether this is a defect in the composition of the material or its subsequent manipulation, or both, is a question in my mind. Plates that have been worn for years have come under my observation which preserved their color remarkably well. Whether these passed through different processes of manufacture, and if so, whether in the preparation of the material or its subsequent treatment, I do not know, but it seemed to indicate conclusively that there was some way of overcoming this difficulty, on which we as yet only occasionally stumbled by accident without comprehending its bearing. The fact of the matter is, we know too little of the material yet to compass all its possibilities. We are groping along blindly in the vestibule of knowledge, credulously accepting the dictum of each new comer as to its proper treatment. What we need is another Wildman to make it the subject of careful, patient, methodical investigation. That we have been able to accomplish so much in comparative ignorance of its properties and behavior under different conditions, indicates a mine rich in possibilities. Rubber was unwieldy and unmanageable in the hands of most, until Prof. Wildman took it, dosed it, drugged it, and finally conjugated it for the profession in all its moods and tenses. The same, I opine, applies to celluloid; we are really only on the threshold of understanding it. The material, both in its preparation and manipulation, must be subjected to patient, thorough, and methodical investigation, its behavior under various conditions studied and analyzed until its whole chapter of possibilities is laid bare; this is what must be done before we can dogmatically assert that celluloid is or is not destined to be the coming base. I think we are perfectly justified in expecting still better things from it, but even in its present crude state it will have a marked influence for good on the profession in developing its taste and rendering less frequent those incongruous cases alluded to in the opening of this paper. With its advent has come the possibility to a great extent, of getting rid of the hateful tyranny of those horribly obstinate block teeth, which so stubbornly refuse to take the hue and impress of surrounding circumstances, and so cramp the skin and taste of the workman. Here then we rejoice in the removal of another obstacle in the grand march of the profession towards the perfect base. This alone if it were its only point of superiority over rubber, would be sufficient to entitle it to consideration and the stamp of approval.

In conclusion then, gentlemen, let us not despair of the future of Mechanical Dentistry. Year after year it has been quietly and steadily fulfilling its mission, and preserved its integrity through all, in spite of the assaults of the ignorant and vicious quacks without, and the abuse of well meaning possibly, but no less persistent Diogenes within. Let us think well of ourselves and our calling, and not fall into the error and injustice of railing at it for the faults and follies of all the irresponsible quacks who may prostitute its livery to their own base uses. If we would have others respect us we must not confound ourselves with such as these. Let us investigate all things with patience and fairness, and not refuse a hearing to that which clamors for our attention, simply because we may be compelled to root out deeply implanted prejudices. This is only putting a drag on the wheels of progress. In a contest between prejudice and egotism, on the one side, and the good of the many on the other, the former are bound to go under. One by one, in spite of ultra-conservatism, the obstacles in the way of perfect work are disappearing, and though still far from perfection, we are slowly but surely working up to it—how slowly and surely depending in a great measure on ourselves.—*Transactions Ill. State Dental Society.*

---

